# Intermediary/purveyor organizations for evidence-based interventions in the US child mental health: characteristics and implementation strategies

**DOI:** 10.1186/s13012-018-0845-3

**Published:** 2019-01-14

**Authors:** Enola Proctor, Cole Hooley, Amber Morse, Stacey McCrary, Hyunil Kim, Patricia L. Kohl

**Affiliations:** 1 Washington University Brown School of Social Work, One Brookings Drive, St. Louis, MO 63130 USA; 2 University of Illinois-Urbana Champaign School of Social Work, Urbana, USA

**Keywords:** Evidence-based intervention, Dissemination, Implementation strategies, Scale-up, NREPP, Child behavior, Health

## Abstract

**Background:**

Many psychosocial interventions are disseminated and supported by organizations, termed “Intermediary/Purveyor Organizations” (IPOs). Because IPOs remain largely unstudied, we lack understanding of their scale and the strategies they utilize. The role and function of organizations that link resource systems with user systems, such as IPOs, have been identified as an important but understudied issue in implementation science. The objectives of this paper are to describe features of IPOs that disseminate evidence-based interventions (EBIs) for child behavioral health and identify the strategies they use to support their implementation.

**Methods:**

The Substance Abuse and Mental Health Services (SAMHSA) National Registry of Evidence-based Programs and Practices (NREPP) listed 119 unique IPOs for the 127 child behavioral health EBIs listed on its website. Data characterizing each organization were drawn from NREPP and GuideStar profiles. From 119 unique IPOs identified, we found contact information for 108. We sent an electronic survey to capture additional organizational information and implementation strategies the IPOs employed in spreading the EBIs; response rate was 50%. Data are presented descriptively and analyzed using ordinary least squares (OLS) regression and Latent Class Analysis (LCA).

**Results:**

Virtually all identified EBIs had an IPO. IPOs train individuals, organizations, and communities and provide supervision for the use of EBIs. About 20% of IPOs trained at large scale, some training 500–1000+ providers annually. IPOs reported using an average of 32 distinct strategies to implement their EBIs, with most using educational, planning, and quality improvement strategies. However, there was little convergence around strategy helpfulness. The only significant predictor of number of strategies used by an IPO was the NREPP-posted implementation readiness score of the intervention. LCA revealed that IPOs either used several implementation strategies or used very few.

**Conclusions:**

Findings add significantly to knowledge about IPO structure, scale, and function. They use numerous and varying implementation strategies but report little consensus in what works. The study advances methods for measuring and characterizing real-world implementation by demonstrating the feasibility of using a common nomenclature, per a published compilation and of LCA for data reduction in characterizing profiles of implementation approaches.

## Background

A recent Institute of Medicine of the National Academies (IOM) [1] report decries the gap between the availability and receipt of evidence-based psychosocial interventions (EBIs) for mental health disorders. Psychosocial EBIs have limited scale; although between 13 and 20% of children and youth experience a mental disorder each year [2], most do not receive EBIs. Bruns et al. report that uptake of mental health treatment for children and youth lags significantly behind adults, with EBIs reaching only 1–3% of youths with serious emotional disturbance [3]. Such data reflect the struggle to scale-up EBI adoption rates to the point where they substantially influence public health on a national scale.

EBI scale-up is limited because of weaknesses in the “distribution system” for bringing treatment discoveries to the attention of practitioners and into practice settings [4]. Unlike drugs and devices, psychosocial interventions have no Federal Drug Administration (FDA)-like delivery system. Instead, distribution is often dependent on the initiative of treatment developers, who lack the resources of an organization such as the FDA. Many treatment developers form organizations to spread information about and provide support for the adoption of their interventions. Such organizations have been called Intermediary/Purveyor Organizations (IPOs) [5, 6]. Well-known IPOs include such international entities as Trauma-Focused Cognitive Behavioral Therapy National Therapist Certification Program, Nurse-Family Partnerships, and Triple P International and their subsidiary, and Triple P America. IPOs have emerged as a major dissemination strategy over the past two decades.

### IPO theory and functions

The term IPO combines two key constructs in the dissemination and implementation literature: purveyor and intermediary. Purveyor has been defined as “an individual or group of individuals representing a program or practice who actively work to implement that practice or program with fidelity and good effect” [5]. Intermediary organizations have been described as “…developing, implementing, and supporting multiple best practice programs or services, as well as building the capacity within an agency or system in order to implement and sustain such programs” [6–8]. Intermediary roles also include consultation, quality assurance, and continuous quality improvement; outcome evaluation; and training [6, 8]. The limited research on these organizations suggests that many organizations identify as both purveyors and intermediaries [6].

The IPO definition and role reflect several key concepts in the implementation science literature, concepts that are widely used but often with overlapping meaning, such as change agency, knowledge broker, or resource system. Many of these terms can be traced conceptually to diffusion of innovation theory [9], but the proliferation of terms may cloud understanding and thwart the harmonization of concepts and data for the field of implementation science.

In order to diffuse, disseminate, and help implement psychosocial interventions, most of these organizations probably engage in many of the core functions of distribution systems. These functions include “identify potential users, promote the product to them, provide them with easy access to the product through convenient (and usually local) channels, allow them to see and use the product before acquiring it, help them acquire it, and support the product after purchase if the buyer has questions or problems [4].”

Many IPOs function to spread particular EBIs, having been established by treatment developers after research to shape the intervention and test effectiveness. Such IPOs bring content expertise in one or two specific interventions to practitioners and agencies. Some such organizations may hold patents or copyrights to the name or training in the EBI. Other IPOs appear to be agnostic toward the specific EBIs they promote. Sometimes labeling themselves “intermediaries,” they often work to find the best intervention fit for a specific organization’s needs, link resources (including perhaps EBI-specific IPOs) to users, and provide coaching about implementation processes that are applicable to implementation of any innovation. In their efforts to find the best intervention fit for a specific organization’s needs, they may help users implement a variety of EBIs.

While specific EBIs have an extensive body of research, a more limited literature describes the role and function of IPOs. See for example literature describing such organizations as the National Center for Nurse-Family Partnerships and Healthy IDEAS [10, 11]. These organizations disseminate and train providers in EBIs for optimizing child development and treating geriatric frailty and depression, respectively. We found only one empirical study of IPOs. Franks and Bory presented survey data reflecting IPO organizational identity, development, activities, and functions [6]. Given the proliferation of terms and the paucity of research focused on IPOs, we lack understanding of their respective functions and the strategies they utilize [12–15]. The dissemination and implementation literature has identified understanding the role and function of organizations who link resource systems with user systems, such as IPOs, as an important but understudied issue [16]. Greater understanding of their structure, sustainability, and operations is needed to help clarify the nature and quality of their contributions to the dissemination and implementation of empirically supported interventions [12].

This paper seeks to shed light on IPOs and the specific strategies they use to implement EBIs in the area of child mental health. We sought to achieve the following objectives:

1. Identify the prevalence of IPOs for spreading child behavioral health EBIs in the US;
2. Identify IPO organizational characteristics;
3. Identify the implementation strategies used by IPOs;
4. Determine the strategies IPOs report as most effective;
5. Explore the association of organizational characteristics with the implementation strategies employed; and
6. Explore a data reduction approach, latent class analysis (LCA), to generate implementation strategy usage profiles.

## Methods

### Sample

The Substance Abuse and Mental Health Services (SAMHSA) National Registry of Evidence-based Programs and Practices (NREPP) served as the sampling frame for this study. NREPP is the federally managed and vetted registry of mental health EBIs and is designed to be a practical decision-making tool to assist clinicians in selecting efficacious treatments [17–20].

All EBIs housed in NREPP have been reviewed and scored by trained experts. Useful information for practitioners is extracted from and uniformly formatted in an easy-to-read report [21]. It is a highly utilized site with 30,052 unique visitors accessing it every month [22]. Though it does not purport to house all existing EBIs, its registry has grown considerably from its initial 25 interventions to a bank of 388 vetted EBIs at the time of our search, June 2015 [21, 22].

Our sample was generated using the advanced search function of NREPP. NREPP is searchable by intervention, rather than by IPO, so the inclusion criteria were based on intervention type and characteristics. Inclusion criteria for the sample: (1) EBI was designated by NREPP as “mental health treatment” or “mental health promotion,” (2) the EBI was designed for children up to age 17, and (3) the EBI’s primary outcomes were mental health related. Mental health-related outcomes included one of the following terms in their outcome description: depression, anxiety, trauma, behavioral disorder, disruptive behavior, substance use, social emotional disorder, aggression, or impulsivity. The resultant pool of EBIs was 127.

A member of the research team examined the EBI profiles to identify the intervention’s IPO. From the 127 EBIs, 119 unique IPOs were identified.

### Data

The first research objective was addressed by reviewing NREPP’s profiles of the EBIs to see which reported having an IPO. We determined whether an EBI had an IPO based on whether or not the NREPP web site listed an organization for the EBI. To address the remaining research objectives, data were obtained from four sources: the IPO’s NREPP profile, the IPO’s GuideStar profile (if the IPO was non-profit), the IPO’s website, and IPO key informant responses to an electronic survey.

A member of the research team extracted data from the aforementioned web-based sources for each IPO. These data included contact information, website, date IPO was established, location, availability of training materials, how training was administered, and availability of provider certification, fidelity tools, and fidelity monitoring. For IPOs with GuideStar profiles, organization financial information was extracted.

We retrieved a Readiness for Dissemination (RFD) score for each EBI from the NREPP website. NREPP assigned each intervention with an RFD score. The scores ranged from 0 to 4, with high scores indicating that dissemination resources are available and are of high quality. RFD was assessed across three domains: availability of implementation materials, availability of training and support resources, and availability of quality assurance procedures. However, as of July 2015, the RFD score no longer included an NREPP review and the scale is no longer used for newly reviewed EBIs [21].

After initial data were gathered about the IPOs, the research team developed an electronic survey in Qualtrics. The research team contacted each IPO to determine who in the organization was most familiar with the IPOs’ implementation practice. Of the 119 IPOs previously identified, contact information was secured for 108. Respondents’ role within their respective IPOs varied. Our initial invitation to participate in the survey asked to speak with someone familiar with the organization’s implementation efforts. Most of the role titles suggested that the respondents were administrators within the IPO (directors, CEO, presidents, directors of training) or researchers involved in the treatment’s development.

The Qualtrics survey was emailed, with up to two reminders. The response rate for fully completed surveys was 50% (*N* = 54). The first section of the survey was designed to capture organizational characteristics not available from the existing web content (e.g., number of staff, specifics on training, cost, and funding).

The second portion of the survey was designed to capture the implementation strategies used by the IPOs. This section was based on the implementation strategy inventory organized by Powell, et al. [23]. The original inventory of 68 strategies was piloted on individuals with expertise in IPO operations within the sphere of child/youth mental health services. The language of certain implementation strategies was modified slightly to fit within the study’s context, and four strategies were excluded that did not apply to the types of strategies these organizations could employ, as determined by the content experts. The final inventory of 64 implementation strategies comprised the second section of the survey. For each item, respondents were asked whether they used or suggested the adopting agency/provider use the listed strategy. In addition, respondents were asked to select up to five strategies they felt were most successful. We did not provide definitions of success. We attempted to reduce social desirability through language stating that no one organization would be expected to use all strategies. Respondents were expected to interpret “success” from their own perspective. We did not ask respondents to identify situations or contexts in which strategies were used or deemed successful.

The responses to these items were used to estimate domain-specific strategy profiles using LCA. Both sections of the survey were tested for usability by individuals with experience working in or with IPOs.

In preparation for the LCA, an additional variable for implementation strategies was created. The individual implementation strategies identified were nested under one of five possible domains: planning, education, financial, restructure, and quality management [23]. To generate an overall implementation score for each of these domains, the number of strategies used in a domain was divided by the number of available strategies in that domain. The denominator for this score was based on the Expert Recommendations for Implementation Change (ERIC) compilation.

These domain-specific scores were then dichotomized into high use and low use, based on a cutoff of the 50th percentile. The resultant variable was a dichotomous variable for each IPO per implementation strategy domain, reflecting either high use or low use of strategies within that domain. These items are utilized in estimating the overall implementation strategy profile by LCA.

### Analysis

Multiple data reduction strategies were used to address our research objectives. The number of EBIs with active contact information for the IPO or its representative was divided by the number of IPOs. We used descriptive statistics to report IPO characteristics. Scores were assigned to the implementation strategies based on the percentage of IPOs who endorsed using the strategy and rating it as most successful to their implementation efforts.

Ordinary least squares (OLS) regression was used as part of an exploratory analysis to study the association between IPO organizational characteristics and number of implementation strategies used. The dependent variable for the regression was the number of implementation strategies used by the IPO. The independent variables included number of IPO staff, the EBI’s NREPP dissemination score, how many years the IPO has been in operation, and the number of clinicians the IPO has trained. The data were assessed to ensure that none of the assumptions of OLS were violated. Regression was executed in Stata v.14.

We used LCA to identify implementation strategy profiles (or latent classes) in the sampled IPOs (*N* = 54). This allowed us to explore whether IPOs varied in meaningful ways in the combination of implementation strategies used. Prior to conducting this analysis, we generated two profile levels. The first was an overall implementation strategy profile based on the use of the domain-level strategies (planning, education, finance, restructure, and quality management). The second was a domain-specific profile based on the use of specific strategies within each domain.

An iterative process was used to determine the optimal number of latent classes based on three different model fit statistics: the likelihood-ratio *G*^2^ statistic, Akaike’s Information Criterion (AIC), and Bayesian Information Criterion (BIC) [24]. A significant result of a likelihood-ratio test suggests that a more complicated model (i.e., “k” class model) has a significantly better model fit than a simpler model (i.e., “k-1” class model) [24]. A lower value on the AIC and BIC suggests a more optimal model in balance between model fit and parsimony [24].

Following the guidelines of methodological experts, selection of the final model took into account these model fit statistics, as well as an attentive examination on model interpretability (i.e., Is each class distinguishable in terms of the pattern of implementation strategies used? Is every class non-trivial in size? Can we define each latent class in a meaningful way?) [24, 25]. We used “PROC LCA” in SAS 9.4 for all LCA [26].

## Results

### Prevalence of IPOs

IPOs were widespread; virtually all of the EBIs appeared to have an IPO, and the majority of them have active contact information (*N* = 108, 90.7%). IPOs represented a substantial financial interest in both the public and private sectors. Those IPOs with GuideStar profiles outlining organizational finances (*N* = 11) showed a collective annual revenue of 1.8 billion.

### IPO characteristics

By nature, IPOs reported that they were training organizations. They train at the individual (85%), organizational (84%), and community levels (33%). IPOs train at a variety of sites: onsite where the EBI will be delivered (89%), at IPO headquarters (58%), and online (47%). To get a sense of IPO training volume, a series of ranges were offered to IPOs for reporting the number of clinicians who completed training. In 2015, the modal range for completing EBI training was 26–50 clinicians. Approximately 11% of IPOs reported a range of 251–500 clinicians who completed training, and 9% of IPOs reported that over 1000 clinicians completed training.

Half of IPOs also offered individual-level certification in their EBI. Slightly over one-quarter offered organization-level certification, and one-tenth offered community-level certification. In order to receive the individual-level certification, 41% of IPOs required ongoing supervision which then provided the IPO with an additional source of revenue.

IPOs reported multiple streams of revenue. Nearly all IPOs (80%) received renumeration from their training efforts. Grants (64%), treatment manual sales (55%), and supervision fees (53%) were other frequently used sources of income.

### Implementation strategies employed

IPOs used an average of 32 (SD = 13.74) distinct strategies in implementing EBIs, reflecting a wide range of approaches. IPOs endorsed a mean of 4.3 of five domains based on the Powell et al. typology [23].

Table 1 (third column) portrays IPOs’ endorsement of particular implementation strategies. By domain, IPOs heavily relied upon provider *educational* strategies, with virtually all distributing educational materials, such as intervention guidelines, manuals, and toolkits. Other frequently employed educational strategies included varying their training methods (90.7%) and materials (88.9%) to facilitate provider learning. Over 85% of IPOs followed training with consultation or booster sessions.

**Table 1:** Percentage of IPOs who use the strategy and rank it within their five most successful

#	Implementation strategy	% of IPOs who use	% of IPOs who listed as top five most successful
Educate domain			
20	Distribute educational materials (e.g., guidelines, manuals, and toolkits) in person, by mail, and/or electronically	96.30	29.63
23	Vary training methods for different learning styles or shape the training to be interactive	90.74	24.07
18	Develop educational materials to make it easier for stakeholders to learn to implement or learn about the EBI	88.89	33.33
26	Provide clinicians with ongoing consultation from an expert in the intervention	88.89	35.19
22	Conduct training in the EBI that included follow-up training, advanced training, booster training, or training to competence	85.19	46.30
21	Conduct educational meetings targeted toward providers, administrators, or other stakeholders to teach them about the EBI	74.07	9.26
24	Use a trained person to educate providers in the adopting organization about the EBI in an effort to change their practice	70.37	5.56
25	Use a train-the-trainer approach	68.52	12.96
30	Use mass media, social media, or promotional materials to reach large numbers of providers or clients the area to spread the word about the intervention	50.00	3.70
33	Encourage educational institutions to train clinicians in the EBI	48.15	1.85
28	Develop a learning collaborative of providers and/or organizations that are also implementing the EBI, and develop ways to learn from one another to foster better implementation	46.30	5.56
29	Have providers shadow other providers who are expert in delivering and implementing the intervention	46.30	3.70
19	Provide a glossary defining key terms related to implementation to promote a common understanding among stakeholders	44.44	3.70
27	Identify certain providers as opinion leaders about the EBI in the hopes that they would influence their colleagues to adopt it	44.44	0.00
32	Attempt to increase demand by influencing the market for the EBI	40.74	1.85
31	Prepare clients to ask questions about available evidence-supported programs/treatments	22.22	0.00
Plan domain			
5	Tailor implementation strategies to address barriers or honor preferences identified through information you had about this agency or the provider(s)	85.19	29.63
10	Engage people who can champion the EBI and spread the word of its need	83.33	35.19
2	Assess the organization’s or the provider’s readiness to implement the EBI, including barriers that may impede implementation, and strengths that could be leveraged for the implementation effort	75.93	29.63
12	Recruit, designate, and train leaders specifically for the changes involved in the adoption/implementation effort	72.22	7.41
14	Encourage adopters to find partners who will share the cost	72.22	5.56
4	Develop a formal implementation plan	70.37	35.19
15	Develop partnerships with organizations that have resources needed to implement the EBI	68.52	11.11
6	Phase implementation, by starting with a small pilot or demonstration project and gradually move to a broader roll-out	66.67	14.81
8	Convene discussions with providers and other stakeholders about the importance of the clinical problems and whether the EBI addresses it appropriately	64.81	1.85
11	Engage clients who might benefit from the EBI in the implementation effort, including advocacy for the implementation	61.11	1.85
9	Involve governing structures, such as a board of directors, in the implementation effort	55.56	3.70
1	Conduct a local needs assessment (collecting and analyzing data related to the need for the EBI in this agency)	53.70	7.41
3	Suggest that the agency visit other sites where a similar implementation effort was considered successful	46.30	5.56
17	Suggest a partnership with a university or academic unit to bring additional skills to the implementation effort	44.44	1.85
16	Obtain, or ask that the agency obtains, written commitments from partners that explicitly stated what they would do to help you implement the EBI	31.48	1.85
13	Suggest mandating use of the EBI	24.07	1.85
7	Suggest or conduct a modeling or simulation of the impact of implementing the EBI prior to implementation	18.52	1.85
Quality management domain			
56	Obtain client feedback on the adoption/implementation effort	77.78	7.41
50	Develop and introduce quality monitoring standards specific to the EBI	74.07	11.11
49	Develop clinical or outcome monitoring procedures for the purpose of quality assurance	70.37	7.41
61	Purposefully re-examine the implementation of the EBI and adjust the implementation strategies as needed	70.37	1.85
64	Capture and share local knowledge about how implementers and clinicians made the EBI work in their setting and then share it with other sites	62.96	3.70
55	Provide clinicians with ongoing supervision in the EBI	61.11	3.70
63	Seek guidance from experts in implementation science (university-affiliated faculty members, quality improvement experts, or implementation professionals)	57.41	1.85
52	Use a centralized technical assistance resource to assist with implementation	51.85	5.56
53	Use audit and feedback (i.e., collecting and summarizing performance data and then giving it to clinicians and administrators) in hopes of changing provider behavior	48.15	3.70
57	Encourage the agency to intervene with clients to increase their adherence to the EBI treatment protocol	44.44	0.00
60	Develop teams of clinicians who are implementing the EBI and encourage the agency to give them protected time to reflect on the implementation effort, share lessons learned, and support one another’s learning	42.59	5.56
59	Involve, hire, and/or consult experts in data management to shape the use of data related to the implementation effort	40.74	0.00
62	Implement cycles of change, small pilots, or plan-study-do-act (PDSA) quality improvements, using small tests of change and refining the implementation based upon what is learned	35.19	3.70
51	Involve advisory boards of different stakeholder groups to oversee implementation	29.63	1.85
54	Develop reminder systems designed to prompt clinicians to deliver the intervention	25.93	0.00
58	Use data warehousing techniques to integrate clinical records across organizations in order to facilitate implementation	16.67	0.00
Finance domain			
39	Encourage the agency to access new funding to facilitate the implementation process	68.52	1.85
41	Work with government or other service payers through requests for proposals (RFPs), to support the delivery of the EBI	48.15	3.70
34	Help the agency develop payment structures to incentivize the adoption of the EBI	25.93	1.85
40	Work with the agency to ensure that the EBI was fully reimbursable	25.93	3.70
42	Suggest that the agency make it easier to bill for services related to the EBI	16.67	0.00
35	Encourage paying providers a set amount per patient for delivering the EBI	9.26	0.00
37	Encourage introduction of other payment schemes such as pre-payment and prospective payment for service, provider salaried service, aligning payment rates with patient outcomes, or removing billing limits	7.41	0.00
38	Encourage the agency to change fees, such that the EBI had lower payment fees	3.70	0.00
36	Encourage the agency to penalize providers financially for failing to deliver the EBI	0.00	0.00
Restructure domain			
43	Shift or revise roles among professionals in order to provide the intervention and eliminate barriers	42.59	0.00
47	Encourage the agency to change the composition of the intervention teams, including different disciplines and skills to make the EBI delivery more likely	37.04	0.00
46	Ask the agency to change record systems to better reflect assessment of implementation of the EBI	31.48	0.00
45	Prompt the use of the EBI by collecting new clinical information from clients and relaying it to the provider	29.63	0.00
44	Increase access to the EBI by suggesting that the agency change the location of service (e.g., co-locating services)	25.93	0.00
48	Change the physical structure or space in the agency (e.g., installing one-way mirrors, making group rooms)	12.96	0.00

Various *planning* strategies comprised the second most frequently endorsed domain. Over 75% of IPOs reported tailoring their work to address agency barriers and facilitators, engaging champions for the EBI, and assessing the readiness for change of the organizations in which providers work. The only other domain with strategies endorsed by 75% or more of IPOs was the *quality* domain. Around three fourths of IPOs developed and introduced quality monitoring tools specific to the EBI, and obtained client feedback on the implementation effort. Two thirds of IPOs endorsed one strategy within the *finance* domain: encouraging agencies to access new funding to facilitate the implementation process. Fewer than half of IPOs endorsed any implementation strategies in the *restructure* domain.

We also determined what percentage of the IPOs’ strategies pertained to each domain (Table 2). Organized by IPO, this table reiterates how the *finance* and *restructure* domains comprised a smaller percentage of each IPOs’ total strategies, with *educational* and *planning* strategies dominating.

**Table 2:** The percentage of the strategies used by domain for each IPO

IPO	Strategies used	Plan (%)	Educate (%)	Finance (%)	Restructure (%)	Quality (%)
1	15	40.0	26.7	6.7	0.0	26.7
2	32	34.4	34.4	6.3	9.4	15.6
3	38	21.1	23.7	15.8	5.3	31.6
4	25	36.0	28.0	4.0	8.0	24.0
5	11	9.1	72.7	0.0	0.0	18.2
6	28	25.0	32.1	10.7	3.6	28.6
7	46	23.9	26.1	15.2	2.2	30.4
8	30	50.0	40.0	3.3	0.0	6.7
9	44	34.1	29.5	6.8	9.1	18.2
10	16	25.0	50.0	12.5	0.0	12.5
11	27	29.6	33.3	0.0	0.0	37.0
12	19	26.3	47.4	0.0	5.3	21.1
13	37	24.3	29.7	2.7	8.1	32.4
14	47	34.0	34.0	6.4	4.3	19.1
15	45	26.7	22.2	15.6	6.7	26.7
16	48	27.1	27.1	4.2	10.4	29.2
17	12	75.0	16.7	8.3	0.0	0.0
18	54	25.9	25.9	11.1	9.3	25.9
19	56	28.6	28.6	8.9	5.4	26.8
20	35	25.7	34.3	0.0	8.6	28.6
21	27	29.6	37.0	3.7	3.7	22.2
22	48	27.1	29.2	6.3	8.3	27.1
23	46	32.6	28.3	8.7	2.2	28.3
24	32	31.3	31.3	6.3	9.4	21.9
25	21	9.5	42.9	9.5	9.5	28.6
26	31	29.0	29.0	6.5	3.2	29.0
27	22	45.5	31.8	4.5	0.0	13.6
28	34	38.2	32.4	0.0	5.9	20.6
29	48	22.9	22.9	8.3	10.4	33.3
30	0	0	0	0	0	0
31	20	30.0	40.0	0.0	5.0	25.0
32	42	26.2	28.6	4.8	9.5	31.0
33	55	27.3	27.3	9.1	9.1	27.3
34	41	29.3	26.8	9.8	7.3	24.4
35	37	29.7	32.4	5.4	5.4	24.3
36	37	32.4	24.3	8.1	2.7	29.7
37	13	15.4	69.2	0.0	0.0	15.4
38	26	26.9	34.6	7.7	0.0	26.9
39	27	29.6	44.4	7.4	0.0	14.8
40	19	26.3	47.4	0.0	0.0	26.3
41	33	39.4	24.2	3.0	0.0	30.3
42	49	26.5	24.5	8.2	8.2	30.6
43	8	0.0	75.0	0.0	12.5	12.5
44	29	27.6	34.5	6.9	0.0	31.0
45	24	33.3	37.5	4.2	12.5	12.5
46	53	24.5	26.4	7.5	11.3	28.3
47	11	27.3	72.7	0.0	0.0	0.0
48	14	21.4	42.9	0.0	0.0	35.7
49	36	33.3	33.3	5.6	2.8	22.2
50	19	26.3	31.6	0.0	0.0	42.1
51	50	32.0	32.0	2.0	6.0	26.0
52	31	35.5	19.4	0.0	9.7	32.3
53	35	37.1	40.0	2.9	0.0	17.1
54	42	28.6	26.2	11.9	7.1	23.8

### Implementation strategies identified as most successful

The last column in Table 1 reflects the percent of IPOs who endorsed a strategy as among the five most successful of the employed strategies. The strategies most frequently endorsed as successful were following training with booster or advanced sessions or training providers to competency (46.3%). Over one third of IPOs endorsed as most successful three additional strategies, two within the *educational* domain—tailoring educational materials and providing ongoing implementation consultation to providers—and one in the *planning* domain—engaging EBI champions.

**Fig. 1:**
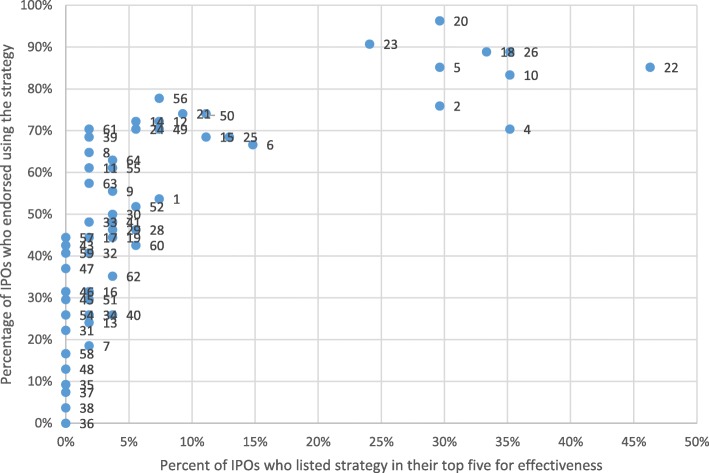
The numbers are the implementation strategies enumerated in Table 1. The numbers are to the right of the dot they are assigned to

Figure 1 plots strategies by the percentage of IPOs who endorsed their usage against the percentage of IPOs who endorsed the same strategy as one of the most successful. The four previously mentioned strategies (#22, #26, #10, #4) comprise the small cluster of strategies that were endorsed by over 30% of respondents. The plot illustrates how there were various strategies endorsed as frequently used and were not endorsed as being one of the most successful.

### IPO characteristics and implementation strategies used

For the exploratory analysis, the results of the multivariate regression indicated that the included covariates explain 27% of the of the variance (*R*^2^ = .2706, *F*(4,46) = 4.27, *p* < .01). The NREPP dissemination readiness score significantly predicted the number of implementation strategies used. Holding all other variables constant, each one point increase on the NREPP score was associated with an IPOs use of approximately eight more implementation strategies.

### Implementation strategy profiles

**Fig. 2:**
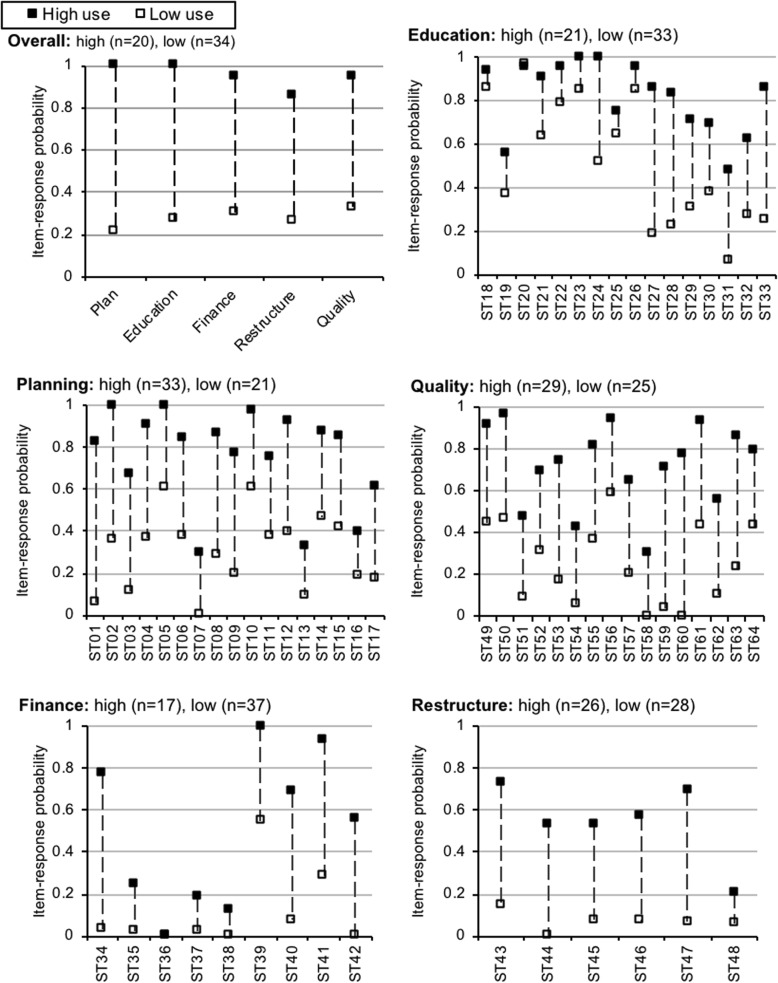
Overall and domain-specific implementation strategy profiles. (ST strategy). Legend: Overall plot. The *x*-axis lists the names of the implementation strategy categories*.* Education, planning, quality, finance, and restructure plots*.* The *x*-axis lists the implementation strategies (ST implementation strategy) per the dimension identified by the plots’ title. And the item response probability for the two groups generated by the latent class analysis. A description of the strategies (identified by their number) can be found in Table 1. The groups are based on the two categories generated by the latent class analysis. They are differentiated by the number of strategies they use. ■ The high-use group. □ The low-use group

Taking into account both the model fit indicators (*G*^2^, AIC, and BIC) and model interpretability, we consistently found that the data supported a two-class model in general. Model fit statistics are presented in Table 2. In all profiles (overall and domain-specific), the two-class model showed high entropy—a measure of certainty in latent class classification, a value closer to one suggesting higher certainty. Based on the pattern in class-based implementation strategy used (Fig. 2), we defined these two classes as the “high-use” group and the “low-use” group.

The class-based use of each implementation strategy (i.e., item-response probabilities) is presented in Fig. 2; fit statistics and other details are found in Table 3. The item response probability represents the likelihood that a strategy (or domain in the overall model) was endorsed by an IPO. For example, in the overall model, 100% of IPOs classified as high-use, endorsed using one or more planning strategies, while only 20% of those in the low-use class did so. For the overall implementation strategy profile, the patterns were quite distinct—high strategy use compared to low strategy use. IPOs either reported a high probability of using strategies within each domain or a low probability of using strategies within a given domain. This suggests that IPOs either used several implementation strategies—across domains—or used very few, with little variability in between.

**Table 3:** Comparison of latent class models for overall implementation strategy profile

			Likelihood ratio difference test					
Model	*G* ^2^	*df*	*G*^2^ difference	*df* difference	*p*	AIC	BIC	Entropy
Overall implementation strategy profile								
2-class	26.27	20	–	–	–	48.27	70.15	.94
3-class	18.67	14	7.60	6	0.2689	52.67	86.49	.76
4-class	14.54	8	4.13	6	0.6591	60.54	106.29	.81
5-class	11.62	2	2.92	6	0.8188	69.62	127.3	.88
Planning strategy profile								
2-class	493.29	131,036	–	–	–	563.29	632.91	.93
3-class	443.57	131,018	49.72	18	0.0001	549.57	654.99	.90
4-class	399.07	131,000	44.5	18	0.0005	541.07	682.29	.97
5-class	405.20	130,982	− 6.13	18	1.0000	583.20	760.22	.97
Education strategy profile								
2-class	447.54	65,502	–	–	–	513.54	579.18	.86
3-class	420.09	65,485	27.45	17	0.0518	520.09	619.54	.88
4-class	399.63	65,468	20.46	17	0.2514	533.63	666.90	.96
5-class	366.65	65,451	32.98	17	0.0113	534.65	701.73	.95
Finance strategy profile								
2-class	55.65	492	–	–	–	93.65	131.44	.88
3-class	38.90	482	16.75	10	0.0801	96.90	154.58	.95
4-class	31.16	472	7.74	10	0.6542	109.16	186.73	.92
5-class	23.84	462	7.32	10	0.6949	121.84	219.30	.95
Restructure strategy profile								
2-class	48.90	50	–	–	–	74.90	100.76	.79
3-class	40.98	43	7.92	7	0.3397	80.98	120.76	.81
4-class	33.14	36	7.84	7	0.3469	87.14	140.84	.89
5-class	20.33	29	12.81	7	0.0769	88.33	155.95	.98
Quality strategy profile								
2-class	472.09	65,502	–	–	–	538.09	603.72	.93
3-class	412.89	65,485	59.2	17	0.0000	512.89	612.34	.96
4-class	379.76	65,468	33.13	17	0.0109	513.76	647.02	.95
5-class	349.16	65,451	30.6	17	0.0223	517.16	684.24	.96

While still maintaining high- vs low-strategy use profiles, some of the domain-specific profiles were more nuanced than the overall profile. In the education domain, for instance, both groups endorsed (1) developing educational materials to make it easier for stakeholders to learn to implement or learn about the EBI; (2) distributing educational materials (e.g., guidelines, manuals, and toolkits) in person, by mail, and/or electronically; ( 3) conducting training in the EBI that included follow-up training, advanced training, booster training, or training to competence; (4) varying training methods for different learning styles, or shape the training to be interactive; and (5) providing clinicians with ongoing consultation from an expert in the intervention. This was not surprising given nearly all IPOs reported using these strategies. Given that 96.34% of IPOs used educational materials (ST20), the actual variance between high- and low-use profiles was nearly non-existent. Conversely, in the finance domain, neither group had high probabilities of using three specific payment approaches (paying providers for delivery of a specific EBI, paying for outcomes, lowering costs of specific EBIs—ST35, ST37, or ST38). Additionally, no IPO in either finance profile reported using ST36, penalties for failing to use EBIs.

### Limitations and strengths

While this paper adds to the limited body of evidence about organizations focused on implementing evidence-based child-focused behavioral health interventions, we acknowledge a number of limitations. First, our analysis focused only on EBIs for child behavioral health. Yet to be explored are the dissemination and implementation activities used by organizations focused on interventions for health, adult behavioral health, child development, and a variety of other issues such as corrections. We drew our IPO list and corresponding readiness scores from NREPP in July 2015; a few months later, NREPP removed the readiness score. NREPP has been critiqued about some of its criteria for intervention inclusion in an “evidence-based” list, with some researchers feeling like the standards are not sufficiently rigorous [27]. Nonetheless, the IOM has noted the importance of NREPP as a central hub to distribute information about mental health EBIs [1] and NREPP is widely accessed and unique in both its detail and scope. Our respondents, key informants of IPOs themselves, reflect the perspectives of only one implementation “actor”—the organizations that exist to roll out EBIs [28]. Results would likely differ if reported from other implementation actors, such as agency directors or policy makers. Furthermore, we acknowledge that our IPO prevalence rates are underestimated due to the difficulty of discerning the existence of certain IPOs from the available contact information on NREPP, coupled with the fact that IPO identification was conducted by one member of our team.

We also acknowledge limitations in measurement. As reflected in prior research [6], capturing scale and cost of IPO activities is challenging, requiring the provision of ordinal ranges (for budget, staff size, providers trained) rather than precise amounts, which we learned that most IPOs cannot retrieve. Measures of organizational characteristics, budgets, and EBI readiness scores were obtained from independent, publicly available sites (and have been used in prior research); however, we do not have data on their validity. We used Powell, an established nomenclature listing and labeling implementation strategies, but respondents’ subjectively determined and reported their strategy use and success with the strategies. While Franks & Bory asked (a convenience sample) of intermediary organization informants to identify their tasks [6], our measurement of dissemination and implementation strategies was based on a published compilation used in studies of implementation in the Veterans Administration [23] but which we adapted to the IPO “actor” perspective. The “number of strategies” employed is a blunt measure. In this work and in the field at large, measurement of implementation strategies is in early stages. However, the sheer number of strategies used seems to matter, given recent findings that use of more strategies is associated with ability to deliver EBIs [29]. Because implementation strategies are generally not available through medical records or archival data, self-report is the current state of art for capturing their use.

Finally, we note that the OLS analysis is exploratory and that our relatively small sample size limits the generalizability of the LCA results. Specifically, it is likely that class results (in our case, two classes) and item-response probabilities would differ using larger samples, particularly given the large number of strategies available. However, we performed the LCA as a proof of concept for a means to examine implementation strategies, and in that regards, we found it to be a useful tool.

## Discussion

EBIs cannot be delivered unless they are adopted by service-providing organizations. How EBIs reach providers, and in turn consumers, remains poorly understood. Thus, our project addresses a troubling gap in the current literature. IPOs operate globally and across the span of healthcare and are particularly important for psychosocial interventions such as behavioral health. Unlike medications, whose adoption is fostered by strategic outreach by the pharmaceutical industry, psychosocial interventions have lacked a centralized distribution source of information or technical assistance.

IPOs are big business. Collectively, with nearly two billion dollars in revenue, these organizations train thousands of clinicians each year. Our own research experience tells us that agency directors considering adoption of new interventions often ask first, “how much will this cost?” Training, training materials, supervision, and certification are the key sources of revenue. Information about the cost of working with an IPO remains difficult to determine, as evidenced by our IPO respondents stating that “it depends.”

Information about implementation strategies, for use by distribution agents such as IPOs or by individual agencies and providers, has only recently been compiled [23, 30]. Strategy compilations inform both implementation research and practice [31]. In 2017, SAMHSA added a new resource to NREPP, the NREPP Learning Center. The Learning Center offers resources to support the selection, implementation, evaluation, and sustainment of evidence-based programs and practices, along with supporting materials in the form of case studies, stories, and videos. A “how to implement” page leads viewers to the Powell et al. compilation of 68 implementation strategies organized into 6 categories which also served as the framework for data collection and analysis in the present study. Regrettably, as of this writing, the future of NREPP and the availability of its resources is unsure [32]. NREPP is no longer maintained. This may hamper the opportunity for providers and organizations to contact IPOs for implementation assistance.

Our results suggest that there is no uniform approach, no magic bullet, to implementation. Nearly all IPOs report using a large number of strategies—nearly three dozens—in their efforts to roll-out EBIs, results aligned with those of Rogal et al. (2017). Moreover, they use strategies in most of the domains identified in the Powell et al. compilation [23]. While many strategies in the Powell compilation were rarely endorsed, we did find some convergence in strategies most frequently used. These include training, formal implementation planning, providing ongoing consultation, engaging local champions, and developing implementation materials.

In contrast to some convergence in strategies used, we found little such convergence around which strategies are most helpful to their work. No single strategy was identified as among the most helpful by more than half of IPOs. Training, which was used by nearly all IPOs, was rated as among the most helpful by only 46% of IPOs. The other most frequently employed strategies were reported as among the most helpful by fewer than 10% of IPOs. This may reflect the complexity of implementing new interventions, or it may reflect that in the absence of knowing what works, implementers use many different approaches. These findings align with those reported by Franks & Bory, indicating that IPOs are engaged in tasks that they do not think are effective [6].

## Conclusions

These findings raise a number of questions for further research. Whether proprietary or not-for-profit, most IPOs employ similar dissemination, training, and support strategies. Research needs to examine the effectiveness of these strategies. Given the high number of strategies used by IPOs, is success a function of the number of strategies used, or do particular strategies contribute disproportionately to success? Recent evidence suggests that adoption of EBIs may indeed be associated with using a high number of strategies. Moreover, successful implementation likely depends on contextual factors and actions by a number of actors. What is the unique contribution of an IPO and what is required from the adopting organization, their staff, and their service recipients? What IPO implementation strategies are effective in which organizational and policy contexts [33]? And why do IPOs report widespread use of implementation strategies in which they have little confidence of success. A priority question for the field is: which strategies are effective, in what context, and for what types of EBIs? Given their preeminent role in implementing evidence-based behavioral health interventions, researchers need to address ways to enhance their effectiveness by advancing understanding of strategy use, measurement, and effectiveness.

## Abbreviations

AIC: Akaike’s Information Criterion
BIC: Bayesian Information Criterion
EBI: Evidence-based interventions
IOM: Institute of Medicine of the National Academies
IPO: Intermediary/Purveyor Organizations
LCA: Latent class analysis
NREPP: National Registry of Evidence-based Programs and Practices
OLS: Ordinary least squares
RFD: Readiness for Dissemination
SAMHSA: Substance Abuse and Mental Health Services

## References

[1] Institute of Medicine (IOM) (2015). Psychosocial interventions for mental and substance use disorders: a framework for establishing evidence-based standards.

[2] Perou R, Bitsko R, Huang L (2013). Mental health surveillance among children--United States, 2005–2011. MMWR Supplements.

[3] Bruns E, Kerns S, Pullmann M, Hensley S, Lutterman T, Hoagwood K (2016). Research, data, and evidence-based treatment use in state behavioral health systems, 2001–2012. Psychiatr Serv.

[4] Kreuter MW, Casey CM, Bernhardt JM. Enhancing dissemination through marketing and distribution systems: a vision for public health: Oxford University Press; 2012.

[5] Fixsen DL, Naoom SF, Blase KA, Friedman RM (2005). Implementation research: a synthesis of the literature.

[6] Franks R, Bory C. Who supports the successful implementation and sustainability of evidence-based practices? Defining and understanding the roles of intermediary and purveyor organizations. New Dir Child Adolesc Dev. 2015;(149):41–56. 10.1002/cad.20112. 10.1002/cad.20112 26375190

[7] Corcoran T, Rowling L, Wise M (2015). The potential contribution of intermediary organizations for implementation of school mental health. Adv School Mental Health Promotion.

[8] Frank RP (2010). Role of the intermediary organization in promoting and disseminating best practices for children and youth: the Connecticut center for effective practice. Emotional Behav Disord Youth.

[9] Rogers EM (1995). Diffusion of innovations, fourth edition.

[10] Olds DL, Hill PL, O’Brien R, Racine D, Moritz P (2003). Taking preventive intervention to scale: the nurse-family partnership. Cogn Behav Pract.

[11] Casado B, Quijano L, Stanley M, Cully J, Steinberg E, Wilson N (2008). Healthy ideas: implementation of a depression program through community-based case management. Gerontologist.

[12] Wynn JR. The role of local intermediary organizations in the youth development field. Chicago: Chapin Hall Center for Children; 2000.

[13] Bullock H, Jaouich A, Lindencrona F, McGraw K, Silvestri F, Vanderpyl J, Watters N. The role of intermediaries in large-scale mental health systems transformation. In: Global Implementation Conference. Dublin; 2015.

[14] Institute of Medicine (IOM) & National Research Council (NRC) (2014). Strategies for scaling effective family-focused preventive interventions to promote children’s cognitive, affective, and behavioral health.

[15] Sholomskas D, Syracuse-Siewert G, Rounsaville B, Ball S, Nuro K, Carroll K (2005). We don’t train in vain: a dissemination trial of three strategies of training clinicians in cognitive-behavioral therapy. J Consult Clin Psychol.

[16] Greenhalgh T, Robert G, Bate P, Kyriakidou O, Macfarlane F, Peacock R (2004). How to spread good ideas: a systematic review of the literature on diffusion, dissemination and sustainability of innovations in health service delivery and organisation.

[17] Aarons G, Hurlburt M, Horwitz S (2011). Advancing a conceptual model of evidence-based practice implementation in public service sectors. Admin Pol Ment Health.

[18] Brounstein P, Gardner S, Backer T (2006). Research to practice: efforts to bring effective prevention to every community. J Prim Prev.

[19] Hennessy K, Finkbiner R, Hill G (2006). The national registry of evidence-based programs and practices: a decision-support tool to advance the use of evidence-based services. Int J Ment Health Addict.

[20] Hennessy K, Green-Hennessy S (2011). A review of mental health interventions in SAMHSA’s National Registry of Evidence-Based Programs and Practices. Psych Serv.

[21] Substance Abuse and Mental Health Services Administration (SAMHSA). National registry of evidence-based programs and practices (NREPP). 2016.

[22] Gillen AC, Elefantis AB, Hodgson AB, Hennessy KD (2013). The international reach of SAMHSA’s National Registry of Evidence-based Programs and Practices. Int J Ment Health.

[23] Powell B, McMillen J, York J (2012). A compilation of strategies for implementing clinical innovations in health and mental health. Med Care Res Rev.

[24] Lanza S, Collins L, Lemmon D, Schafer J (2007). PROC LCA: a SAS procedure for latent class analysis. Struct Equ Modeling.

[25] Muthén B, Muthén L (2000). Integrating person-centered and variable-centered analyses: growth mixture modeling with latent trajectory classes. Alcohol Clin Exp Res.

[26] Lanza ST, Dziak JJ, Huang L, Wagner AT, Collins LM (2015). Proc LCA & Proc LTA users’ guide (Version 1.3.2).

[27] Wright BJ, Zhang SX, Farabee D (2012). A squandered opportunity? A review of SAMHSA’s National Registry of Evidence Based Programs and Practices for offenders. Crime Delinq..

[28] Proctor E, Powell B, McMillen J (2013). Implementation strategies: recommendations for specifying and reporting. Implement Sci.

[29] Rogal S, Yakovchenko V, Chinman M (2017). The association between implementation strategy use and the uptake of hepatitis C treatment in a national sample. Implement Sci.

[30] Powell B, Waltz T, Kirchner J, et al. A refined compilation of implementation strategies: results from the Expert Recommendations for Implementing Change (ERIC) project. Implement Sci. 2015;10:21. 10.1186/s13012-015-0209-1. 10.1186/s13012-015-0209-1 PMC4328074 25889199

[31] Gold R, Bunce A, DeVoe J, et al. Reporting on the strategies needed to implement proven interventions: an example from a ‘real-world’ cross-setting implementation study. Mayo Clin Proc. 10.1016/j.mayocp.2016.03.014. 10.1016/j.mayocp.2016.03.014 PMC4975638 27113199

[32] National Registry of Evidence-based Programs and Practices (NREPP). 2018. Retrieved 18 September 2018 from https://www.samhsa.gov/nrepp. Accessed 18 Sept 2018.

[33] Proctor EK (2017). The pursuit of quality for social work practice: three generations and counting. J Soc Social Work Res.

